# Application of Original Prostate Cancer Progression Model Interacting with Fibroblasts in Preclinical Research

**DOI:** 10.3390/jcm13247837

**Published:** 2024-12-22

**Authors:** Kenichiro Ishii, Kazuhiro Iguchi, Chise Matsuda, Yoshifumi Hirokawa, Yoshiki Sugimura, Masatoshi Watanabe

**Affiliations:** 1 Department of Oncologic Pathology, Mie University Graduate School of Medicine, Tsu 514-8507, Japan; sechico14@med.mie-u.ac.jp (C.M.); ultray2k@med.mie-u.ac.jp (Y.H.); mawata@med.mie-u.ac.jp (M.W.); 2 Department of Nursing, Nagoya University of Arts and Sciences, Nagoya 460-0001, Japan; 3 Laboratory of Community Pharmacy, Gifu Pharmaceutical University, Gifu 501-1196, Japan; iguchi@gifu-pu.ac.jp; 4 Department of Urology, Murase Hospital, Suzuka 513-0801, Japan; sugimura@murase.or.jp

**Keywords:** LNCaP subline, androgen sensitivity, androgen receptor dependency, fibroblasts, growth factors, cytokines

## Abstract

Prostate cancer (PCa) is a heterogeneous disease that exhibits androgen sensitivity and responsiveness to androgen deprivation therapy (ADT). However, ADT induces only temporary remission, and the majority of PCa cases eventually progress to castration-resistant PCa (CRPC). During the development and progression of CRPC, androgen sensitivity and androgen receptor (AR) dependency in PCa cells are often deceased or lost due to ADT or spontaneously arising AR variants even before starting ADT. To prevent CRPC, a clinical PCa model derived from an AR-positive cancer cell line with weak or no androgen sensitivity is required. The human prostate LNCaP cell line is a good model for PCa because of its androgen sensitivity and AR dependency in terms of cell growth and gene expression. Notably, LNCaP cells are heterogeneous cells comprising different clones with natural variations in androgen sensitivity and AR dependency resulting from spontaneously occurring changes. In our group, to obtain androgen-insensitive or weakly sensitive clones spontaneously derived from parental LNCaP cells, we performed a limiting dilution of parental LNCaP cells and obtained several sublines with varying levels of androgen sensitivity and AR dependency. In addition, we established an androgen-insensitive subline from parental LNCaP cells by continuous passage under hormone-depleted conditions. This article provides a unique perspective on our original PCa progression model interacting with fibroblasts and its application in preclinical research.

## 1. Introduction

Clinically, prostate cancer (PCa) is a heterogeneous disease with varying levels of androgen sensitivity and responsiveness to androgen deprivation therapy (ADT). Most patients with early-stage PCa can be treated with appropriate therapies such as radical prostatectomy or irradiation. ADT is the standard systemic therapy given to patients with advanced PCa. Although temporary remission is achieved after ADT, the majority of cases eventually progress to castration-resistant PCa (CRPC), which has a high mortality rate [1].

During CRPC development, androgen sensitivity and androgen receptor (AR) dependency in PCa cells are often decreased or lost due to the negative effects of ADT or spontaneously occurring AR variants before starting ADT [2]. ADT is used for advanced PCa to decrease circulating androgen levels and block AR signaling in PCa cells [3]. Typically, well-differentiated PCa cells are both androgen- and AR-dependent, enabling AR signaling to regulate their cell-cycle progression and differentiation. ADT-induced loss of AR signaling results in AR-independent uncontrolled growth and poor differentiation in PCa cells [4]. Several molecular mechanisms underlying the altered androgen sensitivity and AR dependency of PCa cells have been proposed, such as androgen-independent activation of AR signaling due to AR mutations or altered coactivator levels, as well as activation of alternative growth factor/cytokine pathways [2].

CRPC development after ADT is mediated by different molecular mechanisms, which are classified as adaptation to a low androgen environment due to ADT or clonal selection [5,6,7,8]. Most PCa cells, especially early PCa cells, are sensitive to androgens; however, during disease progression, cell populations differing in androgen sensitivity and AR dependency arise within a tumor cell population. Adaptation of androgen-sensitive PCa cells to a low androgen environment can lead to development of androgen-insensitive PCa cells. In addition, during clonal selection of androgen-insensitive PCa, ADT induces expansion of PCa cells with weak or no androgen sensitivity, which can coexist with androgen-sensitive PCa cells within tumor tissues [5].

In CRPC, several growth factors and cytokines contribute to PCa cell malignancy via AR signaling activation in an androgen-independent manner, which is known as the “outlaw pathway” [9]. In the absence of androgens, epidermal growth factor (EGF), fibroblast growth factor 7 (also known as keratinocyte growth factor), insulin-like growth factor (IGF)-1, and interleukin (IL)-6 can activate AR signaling [10,11,12] via various signaling pathways, including the Akt, signal transducer and activator of transcription 3, and p44/42 mitogen-activated protein kinase (MAPK) pathways [13]. Within the tumor microenvironment (TME), the tumor stroma around PCa cells is enriched in fibroblasts that secrete AR-activating factors, including those mentioned above [14,15].

## 2. History of LNCaP Sublines

A more precise model of clinical PCa requires, at the very least, an AR-positive cancer cell line with weak or no androgen sensitivity. The human prostate LNCaP cell line serves as an optimal PCa model because of its androgen sensitivity and AR dependency in terms of cell growth and gene expression. LNCaP cells are a heterogeneous population of different clones with natural variations in androgen sensitivity and AR dependency induced by spontaneously occurring changes [16,17].

Many studies on CRPC have used the androgen-insensitive AR-negative PCa cell lines PC-3 and DU145. Considering that many androgen-insensitive PCa cases express AR, comparing androgen-sensitive, AR-positive LNCaP cells with PC-3/DU145 cell lines may be irrelevant in terms of acquisition of androgen insensitivity in clinical PCa [18]. PC-3 and DU145 cell lines originated from highly anaplastic tumors from different metastatic sites (bone and brain, respectively) [19,20], and they differ strongly in their aggressiveness from LNCaP cells, which originated from lymph node metastasis [16]. In addition, 22Rv1 cells are also important [21]. The 22Rv1 cell line is derived from a xenograft that was serially propagated in mice after castration-induced regression and relapse of the parental, androgen-dependent CWR22 xenograft. The 22Rv1 cell line expresses AR and prostate-specific antigen (PSA). Its growth is weakly stimulated by DHT and EGF but is not inhibited by TGFβ1.

To develop strategies for the treatment of CRPC, a variety of LNCaP sublines with a range of androgen sensitivity and AR dependency levels have been established and characterized in many laboratories. LNCaP 104R cells, established by long-term androgen ablation in vitro, exhibit androgen hypersensitivity due to elevated AR expression [22]. On the other hand, LNCaP C4 and C4-2 cells, established from LNCaP cells inoculated into castrated mice, exhibit androgen independency [23,24]; these cells also constitutively express PSA in androgen-free medium and are highly metastatic and tumorigenic [25]. Antiandrogen (R)-bicalutamide-resistant LNCaP-Rbic cells, generated after continuous exposure to (R)-bicalutamide, lead to changes in mitochondrial dynamics and to a breakdown in the Drp-1-mediated mitochondrial network [26]. Inflammatory cytokine-resistant, IL-6-positive LNCaP cells, established after prolonged IL-6 treatment, display growth advantages in vitro and in vivo, similar to late-stage PCa cells [27], and these cells are resistant to cytokine-induced apoptosis. Androgen-insensitive LNCaP (AIDL) cells were generated by maintaining parental LNCaP cells in hormone-depleted medium for over 2 years, a condition that mimics hormone ablation therapy [28]. Another LNCaP cell line (LNCaP-cxD) was developed in vitro by culturing LNCaP cells in androgen-depleted medium supplemented with bicalutamide, a condition that simulates combined androgen blockade therapy [29]. In contrast to these LNCaP sublines, LNCaP-r cells were grown in normal medium (androgens present), suggesting that the differences in androgen sensitivity arose from spontaneous generation of, or preexisting, variants in the parental LNCaP cells [30].

**Figure 1:**
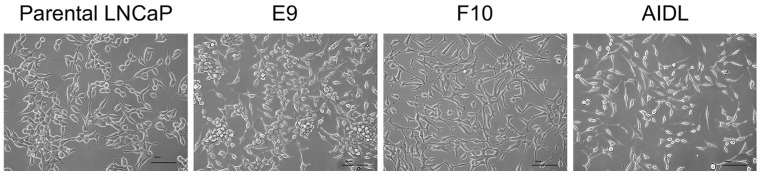
Morphology of LNCaP cell lines. Parental LNCaP cells and two sublines (E9 and F10 cells) were cultured in phenol red (+) RPMI-1640 supplemented with 10% fetal bovine serum (FBS). AIDL cells were cultured in phenol red (−) RPMI-1640 supplemented with 10% charcoal-stripped FBS. Scale bar = 100 µm; magnification, ×200.

To obtain androgen-insensitive or weakly sensitive clones spontaneously generated from parental LNCaP cells, our group performed a limiting dilution of LNCaP cells to obtain several sublines, such as E9 and F10 cells, of LNCaP cells with varying levels of androgen sensitivity and AR dependency (Figure 1) (Table 1) [31,32]. In addition, we established androgen-insensitive AIDL cells from parental LNCaP cells by continuous passaging under hormone-depleted conditions (Figure 1) (Table 1) [28]. AR protein levels were similar among the parental LNCaP cells and the three original sublines E9, F10, and AIDL, and AR-dependent secretion of PSA was detected in LNCaP and E9 cells [15].

## 3. Important Observations

### 3.1. Biochemical Characteristics of LNCaP Sublines

The growth of E9 cells was found to be more rapid in vitro compared with parental LNCaP cells [31]. This may be because of the reduced sensitivity of E9 cells to androgen-related responses, such as growth stimulation and PSA production, compared with parental LNCaP cells. The expression of AR was nearly identical between E9 cells and parental LNCaP cells, suggesting that the difference in androgen sensitivity and AR dependency is not a result of AR expression. We investigated the mechanisms underlying low androgen sensitivity in E9 cells and found that decreased Akt phosphorylation is associated with poor androgen sensitivity [33]. The Akt and p44/42 MAPK pathways are involved in regulation of androgen responses [34,35]. We also demonstrated significantly lower PSA production in parental LNCaP cells after suppression of Akt phosphorylation by PI3K or Akt inhibitors [33]. Thus, E9 cells may be a useful model to evaluate high-grade Gleason tumors with low levels of phosphorylated Akt.

As with E9 cells, F10 cells are also less sensitive to androgen-related responses compared with parental LNCaP cells [32]. The intratumor environment is characterized by low pH, low nutrients, and chronic hypoxia because of poor vascular development [36,37]. In general, parental LNCaP cells die in low-pH/high-nutrient media (pH 6.3/10% FBS) but survive in neutral-pH/low-nutrient media (pH 7.2/0.5% FBS), suggesting that an acidic extracellular environment induces death in these cells. Interestingly, F10 cells can survive under low-pH/low-nutrient conditions, whereas parental LNCaP cells undergo significant cell death under the same conditions. We identified differential expression between parental LNCaP and F10 cells of genes such as BCL2, BIRC5, and DAPK1, which play roles in apoptosis. The full-length AR protein was detectable in all LNCaP sublines (E9, F10, and AIDL) as well as parental LNCaP cells, whereas the AR-V7 protein was detectable only in F10 cells (Table 1) [38]. Thus, we suggest that F10 cells provide a useful model for evaluating the mechanisms underlying their adaptation to a low-pH/low-nutrient environment.

Unlike E9 and F10 cells, AIDL cells are not sensitive to androgen-related responses. Importantly, these cells express AR mRNA and protein, even though PSA expression cannot be induced by androgens [39]. Although mRNA and protein levels were similar, the transcriptional activity of AR in AIDL cells decreased in AIDL cells compared with the parental LNCaP cells, suggesting that AIDL cells exhibit an AR abnormality such as a gene mutation. Parental LNCaP cells possess a point mutation in AR at codon 877 (T877A). In addition to the T877A mutation, we identified a point mutation at codon 741 (W741C) in AIDL cells, suggesting that the T877A/W741C double mutation may cause the androgen insensitivity of AIDL cells [40]. These findings confirm some clinical observations in CRPC [41,42]. No other differences were noted in the AR sequence between parental LNCaP and AIDL cells. In addition, we examined the expression of several AR-co-regulators but did not find any significant differences between parental LNCaP and AIDL cells. Thus, AIDL cells may be useful for evaluating the mechanisms underlying AR mutations in PCa cells.

### 3.2. Responsiveness of LNCaP Sublines to ADT in the Presence or Absence of Stromal Cells

#### 3.2.1. Effects of Paracrine Stromal Stimulation on the Tumorigenesis of PCa Cells Differing in Androgen Sensitivity and AR Dependency

In the TME, aberrant activation between cancer cells and stromal cells significantly contributes to the progression of human cancers including prostate cancer [43,44,45,46,47]. Among stromal cells, activated fibroblasts that stimulate PCa cell proliferation are called carcinoma-associated fibroblasts (CAFs). CAFs lead to malignancy by increasing PCa cell proliferation and invasion and inducing angiogenesis in tumors [48], via secretion of numerous growth factors, cytokines, extracellular matrix (ECM) proteins, and miRNAs that enhance proliferation and invasion in a paracrine manner [49,50]. Recently, Heidegger et al. reported that the ECM collagen composition plays a critical role in PCa progression and has huge potential as a diagnostic and prognostic biomarker and for future therapeutic targeting [51].

Our previous studies demonstrated that LNCaP sublines acquired a more aggressive phenotype in vitro and in vivo compared with parental LNCaP cells [31,32,39,52,53]. In particular, we revealed that tumors derived from the LNCaP sublines E9 and AIDL, but not F10, induced by stromal cells were more developed compared with parental LNCaP tumors (Table 1). Even in the absence of stromal cell stimulation, F10 tumors showed a significant size difference compared with parental LNCaP tumors, whereas E9 and AIDL tumors did not [38,52,53,54].

First of all, we showed no significant difference in tumorigenicity among the three cell lines (parental LNCaP, E9, and AIDL) when grown in the absence of embryonic rat urogenital sinus mesenchyme (UGM) [52,53]. When parental LNCaP, E9, and AIDL cells were grafted alone, the tumorigenic and histological features (e.g., blood spaces) were not affected by the androgen sensitivity status. Microvessel formation was observed in AIDL tumors in the absence of UGM; however, tumor volume did not differ significantly among parental LNCaP, E9, and AIDL tumors, which is considered to be influenced by the blood volume in tumors. On the other hand, in the presence of UGM, tumor growth (especially in size and Ki-67 labeling index) was significantly greater in E9 and AIDL cells than in parental LNCaP cells (Table 1). UGM is composed of undifferentiated fibroblasts that induce instructive and permissive development and differentiation in the prostate [55]. UGM shows androgen-dependent cell growth and produces a number of growth factors and cytokines [53]. For these reasons, UGM is often used for tissue recombination experiments to investigate the influence of paracrine stromal signals [56,57].

Next, we used the commercially available human prostate stromal cell line PrSC as a source of stromal cells to simulate the TME of PCa [58]. E9 tumors grown with PrSC were significantly larger than parental LNCaP tumors grown with PrSC (Table 1). There was no significant difference in tumor size between parental LNCaP tumors and AIDL tumors grown with PrSC. In all PCa cells evaluated (parental LNCaP, E9, and AIDL), cell proliferation (Ki-67 labeling index) was significantly higher in PCa tumors grown in the presence of PrSC compared to in the absence of PrSC. The microvessel density of the tumors was significantly higher in E9 or AIDL cells grafted alone versus in the presence of PrSC.

In other experiments, we investigated the effects of PrSC on the tumorigenesis of F10 cells [32]. Tumors derived from F10 cells implanted into the renal subcapsular space of nude mice in the absence or presence of PrSC stimulation grew more compared with tumors derived from implanted parental LNCaP cells (Table 1). Cell proliferation (according to the Ki-67 labeling index) was also higher in F10 tumors than parental LNCaP tumors. Of interest, F10 tumors were significantly larger than parental LNCaP tumors even in the absence of stromal stimulation.

Finally, we confirmed the effects of paracrine stromal stimulation on tumorigenesis in the parental LNCaP cells and sublines using original fibroblasts derived from patients with PCa [38,54]. As with PrSC, PCa patient-derived fibroblasts increased the tumorigenesis of parental LNCaP, E9, and AIDL cells, but not F10 cells (Table 1). Therefore, our findings suggest that aberrant activation between PCa cells and stromal cells may strongly depend on the specific characteristics of PCa cells.

#### 3.2.2. Effects of ADT on the Tumorigenesis of PCa Cells Differing in Androgen Sensitivity and AR Dependency

ADT is used for advanced PCa to downregulate the concentration of circulating androgens or block the transcriptional activation of AR [3]. Disruption of androgen–AR signaling by ADT may result in deregulated cell-cycle control, which contributes to carcinogenesis [4]. ADT can induce temporary remission, but the majority of treated cases eventually progress to CRPC, which has a high mortality rate [1,59]. Once PCa cells lose their sensitivity to ADT, effective therapies are limited.

Short-term loss of AR function after ADT is associated with apoptosis, reduced PSA secretion, and AR-independent growth of PCa cells (outlaw pathway). Androgen-sensitive and -insensitive interactions between cancer and stromal cells determine the response of PC cells to androgen ablation [53,60]. Selection of good candidates for ADT among advanced PCa patients requires determining the AR dependency of PCa cells interacting with stromal cells before starting ADT. In general, PSA expression is induced via activation of AR signaling by androgens. In our laboratory, Sasaki et al. reported that fibroblasts directly affected PSA expression in parental LNCaP cells cocultured in vitro [54]. In CRPC, the ability of PCa cells to grow in the absence of androgens indicates that AR signaling is activated by CAF-derived growth factors and cytokines rather than androgens. Among these growth factors and cytokines, we confirmed that EGF, IGF-1, and IL-6 directly increase PSA expression in parental LNCaP cells [54], suggesting that fibroblast-derived soluble factors activate AR in the absence of androgens.

**Figure 2:**
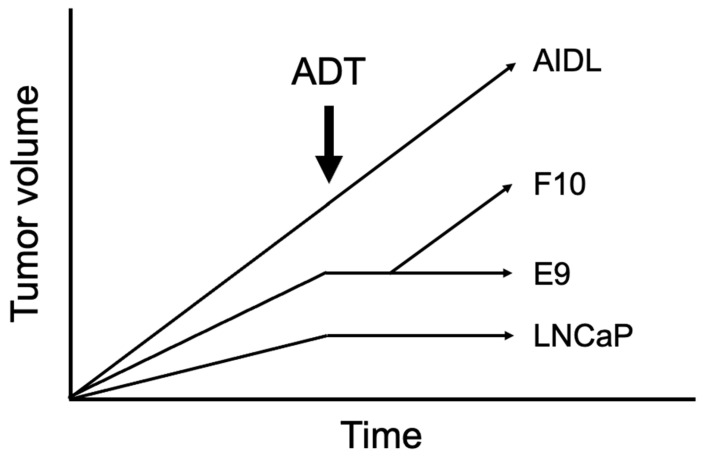
Response to ADT of parental LNCaP cells and three sublines derived from these cells (E9, F10, and AIDL cells).

To investigate the role of fibroblasts in AR activation under ADT, we used three original sublines derived from androgen-sensitive LNCaP cells (E9 and F10 cells, which are weakly androgen sensitive, and AIDL cells, which are androgen insensitive) and fibroblasts derived from PCa patients [38,54]. As shown in Figure 2, the growth of tumors derived from parental LNCaP or E9 cells plus fibroblasts was completely suppressed after ADT; that of tumors derived from F10 cells plus fibroblasts was temporally suppressed after ADT but then increased again; and that of tumors derived from AIDL cells plus fibroblasts was not affected by ADT. Therefore, AR-activating factors secreted from fibroblasts may maintain AR signaling in parental LNCaP and E9 cells after ADT, indicating that these PCa cells can be suppressed by ADT.

### 3.3. Application of LNCaP Sublines in Preclinical Research

#### 3.3.1. Drug Repositioning

Drug repositioning aims to find new indications for existing drugs [61]. In PCa, numerous drug repositioning studies have been performed on non-cancer drugs, including the antidiabetic drug troglitazone (ligand for peroxisome proliferator-activated receptor gamma) [62], the antihypertensive drug candesartan (angiotensin II receptor blocker) [63], naftopidil (a selective α1-adrenoceptor antagonist used to treat benign prostatic hyperplasia) [64], the antiallergy drug tranilast [65], and pirfenidone (PFD; anti-fibrotic and anti-inflammatory drug used to treat idiopathic pulmonary fibrosis) [66]. These drug repositioning approaches help identify new pharmaceutical processes to transform existing drugs into useful sources of new anticancer drugs [67].

We reported that the α1D-selective antagonist naftopidil suppresses the growth of both LNCaP and PC-3 cells by inducing G1 cell-cycle arrest [64]. Using the parental LNCaP cells and sublines, we investigated the biochemical mechanisms of naftopidil in PCa cells, especially the androgen sensitivity and AR dependency of the AR-positive cells and the α1-adrenaline receptor selectivity of naftopidil. In in vitro analyses, naftopidil showed similar growth inhibitory effects on parental LNCaP, E9, and AIDL cells regardless of androgen sensitivity/AR dependency and α1-adrenaline receptor subtype expression (Table 2) [58]. Flow cytometric analysis revealed that naftopidil increased the cell population in the G0/G1 phase but decreased that in the S/G2 phase among E9 cells. Our data demonstrated that the growth inhibitory effect of naftopidil is independent of the androgen sensitivity and AR dependency of the cells or the α1-adrenaline receptor subtype expression in the LNCaP sublines, suggesting that naftopidil induces G1 cell-cycle arrest in various cancer cell types.

In addition, we already reported that PFD treatment suppresses the growth of all PCa cells evaluated (parental LNCaP, E9, F10, AIDL, and PC-3 cells) (Table 2) [66]. In both LNCaP and PC-3 cells, PFD treatment increased the cell population in the G0/G1 phase and decreased that in the S/G2 phase. Among all cells evaluated, greater growth suppression was observed in the parental LNCaP and E9 cells than in the F10 and AIDL cells. TGFβ1 secretion was significantly increased by PFD treatment in all PCa cells, but the increase was greater in parental LNCaP and E9 cells than in F10 and AIDL cells. Our data suggest that PFD-induced growth suppression occurs independently of androgen sensitivity and AR dependency. According to our drug repositioning studies, PFD may provide a novel therapeutic drug that causes G1 cell-cycle arrest in human PCa cells regardless of the androgen sensitivity or AR dependency status.

As a new step of PCa cells’ response to ADT, Li et al. discovered a disruption in the castration-resistant mechanism by inhibiting CDK8 and CDK19 [68]. Mediator kinase inactivation in PCa cells affects stromal gene expression, indicating that Mediator kinase activity in CRPC molded the tumor microenvironment. Thus, the development of Mediator kinase inhibitors is expected as a new class of drugs for the treatment of CRPC.

#### 3.3.2. Cytokine-Induced Progression of PCa Cells

In many cancers, a high serum IL-6 level indicates a poor prognosis, greater metastasis risk, and shorter survival. During ADT, IL-6 affects the malignancy of PCa cells in a ligand (androgen)-independent manner [69,70]. Treatment of LNCaP cells with IL-6 resulted in ligand (androgen)-independent AR activation and induction of neuroendocrine (NE) differentiation [71,72]. Recent evidence has suggested that NE cells and cancer cells with NE features play an important role in the progression of PCa and tolerance to ADT [73].

We observed IL-6-mediated AR activation, including morphological changes such as cell elongation and growth arrest, in not only parental LNCaP but also E9 and F10 cells (Table 2) [15]. The induction of NE differentiation by IL-6 treatment was weak in E9 and F10 cells and absent in AIDL cells. In contrast, IL-6 strongly induced secretion of vascular endothelial growth factor (VEGF), an angiogenic factor, in parental LNCaP and AIDL cells, suggesting that IL-6-induced VEGF secretion is not involved in IL-6-mediated AR activation. These results support the idea that the degree of AR activation in PCa cells is potentially involved in NE differentiation induced by IL-6 treatment.

#### 3.3.3. Cancer-Restraining Effect of Fibroblasts in the TME of PCa

To use primary fibroblasts derived from PCa patients, it is important to consider the patient characteristics, e.g., age, initial PSA, Gleason score, and stage at diagnosis [54,74,75]. In our laboratory, we previously reported that the responsiveness of LNCaP cells to ADT is increased in the presence of fibroblasts that secrete AR-activating factors [54]. Next, we investigated the effects of normal fibroblasts (PrSC) and three fibroblast lines derived from PCa patients (pcPrF-M5, -M28, and -M31) on the expression of cancer-related genes in the parental LNCaP cells and three sublines [76]. The mRNA expression of the tumor suppressor gene *NKX3-1* in parental LNCaP and E9 cells was significantly higher after incubation with conditioned medium from PrSC or prPrF-M5 cells but not that from pcPrF-M28 or pcPrF-M31 cells. *NKX3-1* was not upregulated in F10 or AIDL cells. Among 81 common fibroblast-derived exosomal miRNAs found to have 0.5-fold lower expression in pcPrF-M28 and pcPrF-M31 cells than in PrSC and pcPrF-M5 cells, miR-449c-3p and miR-3121-3p targeted *NKX3-1*. In parental LNCaP cells exclusively, *NKX3-1* mRNA expression was significantly increased after transfection of an miR-3121-3p mimic but not an miR-449c-3p mimic (Table 2). According to these results, fibroblast-derived exosomal miR-3121-3p may be involved in preventing oncogenic dedifferentiation of PCa cells by targeting *NKX3-1* via a mechanism dependent on androgen sensitivity and AR dependency. In addition, our results suggest that visualizing the colocalization of androgen-sensitive and AR-dependent PCa cells, such as LNCaP cells and miR-3121-3p-producing fibroblasts, in PCa tissues may predict the effectiveness of ADT in advance.

## 4. Conclusions

**Figure 3:**
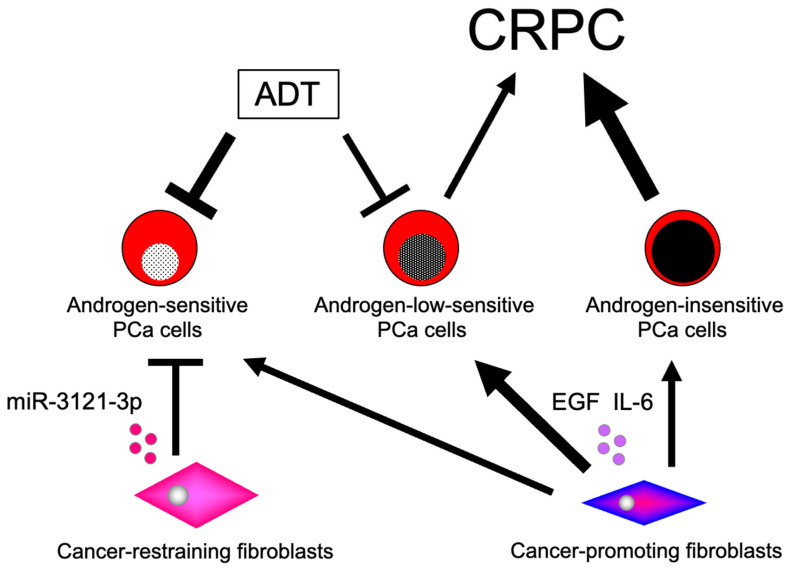
Effects of heterotypic interactions between prostate cancer cells and fibroblasts on the development of resistance to androgen deprivation therapy and progression to castration-resistant prostate cancer. Within the tumor microenvironment, the tumor stroma around PCa cells is enriched in fibroblasts that secret the soluble factors. PCa cells with varying levels of androgen sensitivity and androgen receptor dependency interact with different types of fibroblasts, leading to the development of resistance to ADT and progression to CRPC. ADT: androgen deprivation therapy; CRPC: castration-resistant prostate cancer; PCa: prostate cancer.

Most PCa cells, especially early-stage cells, display androgen sensitivity. During disease progression, however, the tumor cells eventually differ in their androgen sensitivity and AR dependency. To prevent the development and progression of CRPC, we hypothesize that preservation of AR signaling after ADT is essential. Using parental LNCaP cells and sublines derived from these cells, we provide the following ideas (Figure 3). First, to treat certain PCa cells (e.g., E9 cells), fibroblasts that secrete AR-activating factors should be maintained because of the preserved AR signaling in PCa cells after ADT. Second, ADT efficacy may be limited in PCa cells expressing AR-V7 (e.g., F10 cells). Third, in androgen-insensitive PCa cells (e.g., AIDL cells), ADT has no effect because of the AR independence of these cells.

Before starting this treatment, clinically established molecular biomarkers to predict the effectiveness of ADT need to be identified. In the near future, determining the biochemical characteristics of PCa cells differing in androgen sensitivity and AR dependency and evaluating the histopathological characteristics of various types of PCa cells in tumor tissues may help predict the effectiveness of ADT.

## Figures and Tables

**Table 1:** Biochemical characteristics of LNCaP cell lines.

LNCaP Cell Line	Androgen Sensitivity	AR Dependency	Expression of AR-V7	Responsiveness to Stromal Cells
Parental LNCaP	High	High	N.D.	Yes
E9	Low	Low	N.D.	Yes
F10	Low	None	Yes	No
AIDL	None	None	N.D.	Yes

Low/High: low/high androgen sensitivity or AR dependency compared with parental LNCaP cells (High). No/Yes: absent/present AR-V7 expression or stromal cell responsiveness compared with parental LNCaP cells (Yes). AR, androgen receptor; AR-V7, androgen receptor splice variant 7; N.D., not detected.

**Table 2:** Effects of biochemical characteristics of LNCaP cell lines on responsiveness to drugs, cytokine, and miRNA.

LNCaP Cell Line	Growth Inhibition		NE Differentiation	Cancer-Restraining
	Naftopidil	PFD	IL-6	miR-3121-3p
Parental LNCaP	Yes	Yes	Yes	Yes
E9	Yes	Yes	Yes	No
F10	N/A	Yes	Yes	No
AIDL	Yes	Yes	No	No

No/Yes: absent/present growth inhibition, NE differentiation, or cancer-restraining effects compared with parental LNCaP cells (Yes). NE, neuroendocrine; PFD, pirfenidone; N/A, not applicable.
